# Mind the Noise When Identifying Computational Models of Cognition from Brain Activity

**DOI:** 10.3389/fnins.2016.00573

**Published:** 2016-12-27

**Authors:** Antonio Kolossa, Bruno Kopp

**Affiliations:** Department of Neurology, Hannover Medical SchoolHannover, Germany

**Keywords:** computational modeling, functional brain imaging, event-related potentials, signal-to-noise ratio, validity, model identifiability, design optimization

## Abstract

The aim of this study was to analyze how measurement error affects the validity of modeling studies in computational neuroscience. A synthetic validity test was created using simulated P300 event-related potentials as an example. The model space comprised four computational models of single-trial P300 amplitude fluctuations which differed in terms of complexity and dependency. The single-trial fluctuation of simulated P300 amplitudes was computed on the basis of one of the models, at various levels of measurement error and at various numbers of data points. Bayesian model selection was performed based on exceedance probabilities. At very low numbers of data points, the least complex model generally outperformed the data-generating model. Invalid model identification also occurred at low levels of data quality and under low numbers of data points if the winning model's predictors were closely correlated with the predictors from the data-generating model. Given sufficient data quality and numbers of data points, the data-generating model could be correctly identified, even against models which were very similar to the data-generating model. Thus, a number of variables affects the validity of computational modeling studies, and data quality and numbers of data points are among the main factors relevant to the issue. Further, the nature of the model space (i.e., model complexity, model dependency) should not be neglected. This study provided quantitative results which show the importance of ensuring the validity of computational modeling via adequately prepared studies. The accomplishment of synthetic validity tests is recommended for future applications. Beyond that, we propose to render the demonstration of sufficient validity via adequate simulations mandatory to computational modeling studies.

## 1. Introduction

Computational biology involves mathematical modeling techniques to study biological systems. For example, computational neuroscience is the study of brain function in terms of quantitative models of information processing in the nervous system (e.g., Sejnowski et al., 1988). Computational cognitive neuroscience (CCN) represents an emerging subfield of computational neuroscience which aims to identify computational models of cognition from measures of brain activity (e.g., Knill and Pouget, 2004; Friston, 2005; O'Reilly et al., 2012, 2013; Koechlin, 2014; Gerstner and Frémaux, 2015; Kira et al., 2015; Pecevski and Maass, 2016). The general framework of CCN rapidly gains popularity because of both its overwhelming advantages compared to non-computational methods and its potential clinical importance. The emergence of new fields, such as for example computational psychiatry (e.g., Montague et al., 2012; Corlett and Fletcher, 2014; Stephan and Mathys, 2014; Adams et al., 2016; Huys et al., 2016), may serve as an indicator of these developments. Still another reason why CCN rapidly gains popularity is that it represents a modality-general approach in that it deals with all functional brain imaging modalities such as, for example, functional magnetic resonance imaging (fMRI), magnetoencephalography (MEG), and electroencephalography (EEG).

CCN includes many techniques for computational modeling, i.e., for analyzing relationships between observed data and latent variables. The common approach is *forward* modeling which expresses the observed data as functions of some predictors. Since forward models provide a model for the generation of the observed data, they are also referred to as generative models in the machine learning literature (Haufe et al., 2014). In contrast, *backward* models “extract” latent variables as functions of the observed data, i.e., they reverse the direction of the functional dependency between latent variables and data compared to forward models. They are typically used if there is no need to model the generation of the data, i.e., when one is only interested in transforming observed data into a (potentially low-dimensional) representation in which they exhibit certain desired characteristics (a familiar example is brain-computer interfaces; e.g., Nicolelis, 2001; Blankertz et al., 2007).

In this article, we present a simulation study evaluating methodological issues related to the validity of forward modeling studies. Validity implies in the context of CCN that a forward modeling study renders it possible to identify from observed brain activities the proper generative model which usually corresponds to a computational model of cognition. A major threat to the validity of forward modeling studies lies in the fact that observed brain activities are a combination of many sources of variation some of which are systematically related to the latent variables (i.e., the desired signal), but some of which represent measurement error and background noise. Our study is the first to handle the noise problem as a validity problem of identifying the proper data-generative model.

We recognized that the effects of the signal-to-noise ratio (SNR) on the validity of forward modeling studies are still not dealt with in the literature. In order to fill that gap, our study used synthetic EEG data of varying data quality (i.e., degrees of noise) to examine the circumstances under which the proper, rather than an improper, generative model can be identified, thereby providing formal insights into the relationship between variations in data quality and the validity of forward modeling studies.

We chose single-trial P300 (or P3, also P3b) amplitudes of the event-related brain potential (ERP) mainly for two reasons as an example. First, it is well-established that single-trial P300 amplitudes are sensitive to the degree to which eliciting stimuli are surprising (Donchin, 1981), and surprise is a well-defined information theoretic metric (Shannon and Weaver, 1948). Second, the relevant model space is comparatively limited: Squires et al. (1976) presented a model of P300 amplitude fluctuations, based on the concept of expectancy, see below. Basically following that lead, we suggested a computational model (Kolossa et al., 2013) which represents a refinement of Squires et al. (1976). An alternative to these two multifactorial computational models was proposed by Mars et al. (2008) who suggested a simple unifactorial computational model that keeps track of the relative frequency of stimuli.

We chose our own model (Kolossa et al., 2013) as the generative model of the synthetic EEG data, and we analyzed whether Bayesian model comparison techniques (Friston et al., 2007; Stephan et al., 2009; Penny, 2012)—which are commonly used for model selection of ERP data (Mars et al., 2008; Ostwald et al., 2012; Kolossa et al., 2013, 2015; Lieder et al., 2013)—were capable to identify the proper generative model under varying degrees of noise. For sake of simplicity, we consider single-channel EEG data (i.e., single-trial amplitude measures obtained from one single recording channel) rather than multichannel EEG data. Note that multivariate methods combine information from different channels and thus render it possible to cancel out some degree of noise (Makeig et al., 1996; Haufe et al., 2014). However, the application of multivariate methods does not solve, but merely ameliorates the noise problem.

## 2. Materials and methods

### 2.1. System model

Single-trial ERPs can be extracted from the EEG and they are measurable traces of cognitive processes (Luck, 2014). Here, we used synthetic ERPs that have the advantage of providing well-defined observables, i.e., data = signal + noise. Thus, this study explores the framework of CCN by starting with known signals and by adding various levels of noise in order to see whether the signal-generating model can be re-established against a background of alternative models (see below for details). We are mainly interested to see how the validity of the model selection hinges upon (a) the SNR ratio, (b) the number of data, (c) the dependency in the model space, and (d) model complexity.

Three related models of single-trial P300 amplitude fluctuations are taken from the literature, namely the SQU model proposed by Squires et al. (1976), the MAR model proposed by Mars et al. (2008), and the DIF model published in Kolossa et al. (2013). These models, along with the null model (NUL), constitute a model space M={NUL,MAR,SQU,DIF}. A series of *N* random events *k* = {1, …, *K*}, here with *K* = 2, is drawn to form observations *o*(*n*) = *k*, with trial index *n* ∈ {1, …, *N*}. The DIF model (see below) is used to calculate the surprise *I*_*P*_(*n*) over the observation *o*(*n*) = *k*. An offset ϑ is added to *I*_*P*_(*n*) to yield the signal *s*(*n*)

(1) $$\begin{array}{l} s\left(n\right)=\vartheta +I_{P}\left(n\right). \end{array}$$

Artificial noise ϵ(*n*) is then added to *s*(*n*) to yield the synthetic ERP (sERP) *y*(*n*) following

(2) $$\begin{array}{l} y\left(n\right)=s\left(n\right)+\epsilon \left(n\right). \end{array}$$

This procedure is repeated for *L* = 16 virtual subjects ℓ = {1, …, *L*}. Random effects Bayesian model selection for group studies (Stephan et al., 2009) is then used to evaluate which of the models m∈M actually generated the ERP sequence *I*_*P*_(*n*). These analyses are repeated for different levels of noise and various numbers of data points *N*.

In the following, Bayesian model selection (BMS) is shortly introduced before the model space is formally defined. An introduction to SNR estimation for ERPs precedes the detailed description of the analysis framework. A short note on notation: small bold symbols refer to vectors, capital bold symbols to matrices, and []^*T*^ denotes the transpose. Thus, the vector **y** = [*y*(*n* = 1), …, *y*(*n* = *N*)]^*T*^ captures the synthetic data over trials, while **ϵ** = [ϵ(*n* = 1), …, ϵ(*n* = *N*)]^*T*^ represents the corresponding noise. All simulations were performed using MATLAB 7.11.0 and the Statistical Parametric Mapping (SPM8) software.

### 2.2. Bayesian model selection

BMS methods are widely applied in many fields (Raftery, 1995; Hoeting et al., 1999; Penny and Roberts, 2002; Pitt and Myung, 2002; Beal and Ghahramani, 2003; Kemp et al., 2007; Hoijtink et al., 2008; Vyshemirsky and Girolami, 2008; Toni et al., 2009; Penny et al., 2010; Kolossa et al., 2016). We used a two-level hierarchical general linear model (GLM) with the Parametric Empirical Bayesian (PEB) scheme and random effects BMS for group studies as implemented in the SPM software (Friston et al., 2002, 2007; Stephan et al., 2009). The two-level hierarchical model equips a standard general linear model with a second level that places constraints on the parameter estimates of the first level. For each subject ℓ and model *m* of the model space M, the log-evidence is approximated with a variational free energy bound *F*_*m*, ℓ_ which consists of an accuracy and a complexity term (Penny et al., 2004; Friston et al., 2007; Penny, 2012). Random effects (RFX) BMS for group studies computes exceedance probabilities φ_*m*_ for all models, which equals the probability that model *m* is more likely than all other models (Stephan et al., 2009).

The two-level GLM is of the form

(3) $$\begin{array}{r} \text{y}={\text{X}}^{\left(1\right)}\theta +\epsilon^{\left(1\right)}\\ \theta =\epsilon^{\left(2\right)}. \end{array}$$

The first level of the GLM contains two parameters $\theta ={\left[\theta_{1}\;\theta_{2}\right]}^{T}$ which model intercept and slope, respectively. The model-dependent compositions of the first-level design matrices **X**^(1)^ will be shown in the model-specific sections below. The second level of the GLM sets an unconstrained prior on the first-level parameters **θ** and allows for single-level Bayesian inference (Ostwald et al., 2012).

All errors are assumed to be normally distributed with $\epsilon \left(1\right)\sim N\left(0,\Sigma_{\epsilon }^{\left(1\right)}\right)$ and $\epsilon \left(2\right)\sim N\left(0,\Sigma_{\epsilon }^{\left(2\right)}\right)$. The covariance is parameterized following $\Sigma_{\epsilon }^{\left(1\right)}=\lambda^{\left(1\right)}{\text{I}}_{N}$ and $\Sigma_{\epsilon }^{\left(2\right)}=\lambda^{\left(2\right)}{\text{I}}_{2}$, with ${\text{I}}_{N}\in {\mathbb{R}}^{N\times N}$ being an identity matrix. The parameters **θ** and the hyper-parameters λ^(1)^ and λ^(2)^ are estimated using an expectation maximization (EM) algorithm. After convergence of the EM, the conditional means of the first-level parameters **μ**_θ|**y**_ are used as point estimates (Friston et al., 2002) for model fitting to yield the sERP estimates

(4) $$\begin{array}{l} \hat{\text{s}}={\text{X}}^{\left(1\right)}\mu_{\theta |\text{y}} \end{array}$$

before calculation of the Spearman correlation and the explained variance (see below).

### 2.3. Spearman correlation

We use the Spearman correlation ρ as a measure of similarity between two models *m* = 1 and *m* = 2. It follows

(5) $$\begin{array}{l} \rho =1-\frac{6\sum_{n=1}^{N}d^{2}\left(n\right)}{N\left(N^{2}-1\right)}, \end{array}$$

with *d*(*n*) being the distance between the ranks of the sERP predictors from two models ŝ_*m* = 1_(*n*) and ŝ_*m* = 2_(*n*) on trial *n*.

### 2.4. Explained variance

As an absolute measure of fit of the models to the data, we use the explained variance calculated as the squared correlation coefficient

(6) $$\begin{array}{l} R^{2}={\left(\frac{\sum_{n=1}^{N}\left(ŝ\left(n\right)-\bar{ŝ}\right)\left(y\left(n\right)-\bar{y}\right)}{\sqrt{\sum_{n=1}^{N}{\left(ŝ\left(n\right)-\bar{ŝ}\right)}^{2}\sum_{n=1}^{N}{\left(y\left(n\right)-\bar{y}\right)}^{2}}}\right)}^{2}, \end{array}$$

with $\bar{ŝ}$ and $\bar{y}$ as the means of $\hat{\text{s}}$ and **y**, respectively.

### 2.5. Model space

This section details the four models which constitute the model space M={NUL,MAR,SQU,DIF}. It also specifies the respective first-level design matrices **X**^(1)^ which are input to the model estimation and selection framework. For all models except for the NUL model, the first-level design matrices consist of a constant term and the respective ERP predictor, as will be detailed below.

#### 2.5.1. NUL model

The NUL model represents the null hypothesis that the signal is constant and variation in the data is solely due to noise. Thus, the first level design matrix is an all one column vector

(7) $$\begin{array}{l} X^{\left(1\right)}=x^{\left(1\right)}=\left[\begin{array}{c} 1\\ \vdots \\ 1 \end{array}\right]\in {\mathbb{R}}^{N}. \end{array}$$

Notice that the GLM (3) for the NUL model consists only of an intercept θ_1_, thus greatly reducing the complexity of this model.

#### 2.5.2. MAR model

The MAR model as proposed by Mars et al. (2008) uses predictive surprise *I*_*P*_(*n*) over observations to predict the sERP. This model keeps track of the observation probability *P*_*k*_(*n*) according to

(8) $$\begin{array}{l} P_{k}\left(n\right)=\frac{{\tilde{c}}_{L,k}\left(n\right)+1}{\left(n-1\right)+K}, \end{array}$$

with the long-term memory count function ${\tilde{c}}_{L,k}\left(n\right)$ counting the number of occurrences of event *k* until trial *n*−1. Please refer to Mars et al. (2008) or Kolossa et al. (2013) for further details on the count function. After an observation is made, the observation probability is transformed to predictive surprise following

(9) $$\begin{array}{l} I_{P}\left(n\right)=-\log_{2}\left(P_{k\;=\;o\left(n\right)}\left(n\right)\right). \end{array}$$

The first-level design matrix for the MAR model has the form

(10) $$\begin{array}{l} X^{\left(1\right)}=\left[\begin{array}{cc} 1 & I_{P}(n\text{​}=\text{​}1)\\ \vdots & \vdots \\ 1 & I_{P}(n\text{​}=\text{​}N) \end{array}\right]\in {\mathbb{R}}^{N\times 2}, \end{array}$$

thus modeling the sERP to be composed of an offset as in (1) and predictive surprise.

#### 2.5.3. SQU model

The SQU model uses expectancy *E*_*k*_(*n*) for event *k* ∈ {1, 2} on trial *n* as sERP predictor. While Squires et al. (1976) originally did not provide a complete analytical form of their model, Kolossa et al. (2013) present a thoroughly mathematical reformulation of their approach. The expectancy that event *k* ∈ {1, 2} will be observed on trial *n* ∈ {1, …, *N*} consists of an exponentially decaying count function for short-term memory, ${\overset{⌣}{c}}_{\text{S},k}\left(n\right)$, a count function for alternation expectancy, ${\overset{⌣}{c}}_{\text{A},k}(n)$, along with the global event probability, *P*_*k*_, which combine to

(11) $$\begin{array}{l} {\text{E}}_{k}(n)=0.235\;\cdot \;{\overset{⌣}{c}}_{\text{S},k}(n)+0.033\;\cdot \;{\overset{⌣}{c}}_{\text{A},k}(n)+0.505\;\cdot {\text{ P}}_{k}-0.027. \end{array}$$

The constants are empirically derived best-fitting parameters. The interested reader is referred to Kolossa et al. (2013) for a detailed derivation of the count functions ${\overset{⌣}{c}}_{\text{S},k}\left(n\right)$ and ${\overset{⌣}{c}}_{\text{A},k}(n)$. Analogously to the MAR model, the first-level design matrix for the SQU model is of the form

(12) $$\begin{array}{l} X^{\left(1\right)}=\left[\begin{array}{cc} 1 & {\text{E}}_{k\;=\;o(n)}(n\text{​}=\text{​}1)\\ \vdots & \vdots \\ 1 & {\text{E}}_{k\;=\;o(n)}(n\text{​}=\text{​}N) \end{array}\right]\in {\mathbb{R}}^{N\times 2}. \end{array}$$

#### 2.5.4. DIF model

The digital filtering (DIF) model predicts the sERP with predictive surprise akin to the MAR model. It keeps track of the observation probability *P*_*k*_(*n*) but with an exponentially decaying short-term memory count function, *c*_*S, k*_(*n*), an alternation expectation contribution, *c*_*A, k*_(*n*), and exponentially decaying long-term memory count function, *c*_*L, k*_(*n*). It thus combines properties of the SQU and MAR model. The three contributions and an additive probability-normalizing constant $\frac{1}{C}$ combine to

(13) $$\begin{array}{l} P_{k}\left(n\right)=0.83\cdot c_{L,k}\left(n\right)+0.12\cdot c_{S,k}\left(n\right)+0.05\cdot \left[c_{A,k}\left(n\right)+\frac{1}{C}\right]. \end{array}$$

The interested reader is referred to Kolossa et al. (2013) for details on the count functions and the empirical derivation of the constants. Once event *k* on trial *n* has been observed, the observation probability is transformed to predictive surprise (9), yielding the first-level design matrix

(14) $$\begin{array}{l} X^{\left(1\right)}=\left[\begin{array}{cc} 1 & I_{P}(n\text{​}=\text{​}1)\\ \vdots & \vdots \\ 1 & I_{P}(n\text{​}=\text{​}N) \end{array}\right]\in {\mathbb{R}}^{N\times 2}, \end{array}$$

akin to the MAR and SQU models.

### 2.6. Signal-to-noise ratio (SNR)

Though often neglected, the SNR (power) ratio of the EEG defines the boundary conditions in ERP research. It depends on the SNR how many trials are necessary for meaningful ERP estimates (Luck, 2004) and reliable BMS (Penny, 2012). Early methods for SNR estimation for ERPs go back to the times of the discovery of the P300 (Sutton et al., 1965; Schimmel, 1967). Only few approaches were presented in later years (Coppola et al., 1978; Başar, 1980; Raz et al., 1988; Puce et al., 1994). The one proposed by Möcks et al. (1988) is still used as the basis for current developments (beim Graben, 2001; Paukkunen et al., 2010).

#### 2.6.1. Estimating the SNR

We follow the approach from Möcks et al. (1988) which we now briefly describe. Notice that the SNR is calculated for each event type *k* separately and averaged afterwards. So first, the sERP amplitudes *y*(*n*) are separated according to their event type *k*, yielding *y*_*k*_(*n*), with *n* ∈ {1, …, *N*_*k*_} and *N*_*k*_ as the total number of trials in which event *k* was observed. The sERPs *y*_*k*_(*n*) are assumed to be composed of the signal *s*_*k*_ and stationary ergodic noise ϵ_*k*_(*n*) with variance $\sigma_{\epsilon_{k}}^{2}$ (beim Graben, 2001), yielding

(15) $$\begin{array}{l} y_{k}\left(n\right)=s_{k}+\epsilon_{k}\left(n\right), \end{array}$$

with *s*_*k*_ as constant over trials. These assumptions are not met for real ERP amplitudes, but they are nevertheless accepted as useful simplifications (Möcks et al., 1988). The SNR for event *k* is defined as the ratio of the power of the signal over the noise power (beim Graben, 2001)

(16) $$\begin{array}{l} {\text{SNR}}_{k}=\frac{P_{s_{k}}}{P_{\epsilon_{k}}}=\frac{s_{k}^{2}}{\sigma_{\epsilon_{k}}^{2}}. \end{array}$$

Möcks et al. (1988) propose the noise power estimate

(17) $$\begin{array}{l} {\hat{P}}_{\epsilon_{k}}=\frac{1}{N_{k}-1}\overset{N_{k}}{\underset{n=1}{\sum }}{\left(y_{k}\left(n\right)-{\bar{y}}_{k}\right)}^{2}, \end{array}$$

with

(18) $$\begin{array}{l} {\bar{y}}_{k}=\frac{1}{N_{k}}\overset{N_{k}}{\underset{n\;=\;1}{\sum }}y_{k}\left(n\right). \end{array}$$

The power of the sERP follows (beim Graben, 2001)

(19) $$\begin{array}{l} P_{y_{k}}=\bar{y_{k}^{2}}=\frac{1}{N_{k}}\overset{N_{k}}{\underset{n\;=\;1}{\sum }}y_{k}^{2}\left(n\right) \end{array}$$

and, assuming statistical independence between signal and noise, it is composed of the power of the signal *P*_*s*_*k*__ and the power of the noise *P*_ϵ_*k*__ according to

(20) $$\begin{array}{l} P_{y_{k}}=P_{s_{k}}+\frac{1}{N_{k}}P_{\epsilon_{k}}. \end{array}$$

Notice that the noise left in *P*_*y*_*k*__ is attenuated by the factor *N*_*k*_, therefore the scaling of the noise power *P*_ϵ_*k*__ by $\frac{1}{N_{k}}$ in (20) (Möcks et al., 1988; Paukkunen et al., 2010; Czanner et al., 2015). The signal power can now be estimated from (20)

(21) $$\begin{array}{l} {\hat{P}}_{s_{k}}=P_{y_{k}}-\frac{1}{N_{k}}{\hat{P}}_{\epsilon_{k}} \end{array}$$

and the SNR estimate ${\text{S}\hat{\text{N}}\text{R}}_{k}$ in [dB] follows

(22) $$\begin{array}{ll} {\text{S}\hat{\text{N}}\text{R}}_{k}\left[\text{dB}\right]=10\log_{10}\frac{{\hat{P}}_{s_{k}}}{{\hat{P}}_{\epsilon_{k}}}. & \;\left(22\right) \end{array}$$

#### 2.6.2. Setting the SNR

We employ the SNR estimation methods described above for generating sERPs with a specific SNR. Notice that even in response to the same event type *k*, real ERPs are not constant over trials (Squires et al., 1976; Mars et al., 2008; Ostwald et al., 2012; Kolossa et al., 2013, 2015; Lieder et al., 2013). In order to make this work applicable to real ERPs we use a trial-variable signal *s*_*k*_(*n*) in (15) instead of a constant *s*_*k*_. The signal power then follows

(23) $$\begin{array}{l} P_{s_{k}}=\frac{1}{N}\overset{N}{\underset{n=1}{\sum }}s_{k}^{2}\left(n\right) \end{array}$$

and the noise power is known as

(24) $$\begin{array}{l} P_{\epsilon_{k}}=\sigma_{\epsilon_{k}}^{2}. \end{array}$$

Inserting (24) in (22) and solving for $\sigma_{\epsilon_{k}}^{2}$ yields

(25) $$\begin{array}{l} \sigma_{\epsilon_{k}}^{2}=\frac{P_{s_{k}}}{10^{\frac{\text{SNR }[\text{dB}]}{10}}} \end{array}$$

which is the sought after error variance $\sigma_{\epsilon_{k}}^{2}$ for a desired SNR [dB], given *s*_*k*_(*n*).

### 2.7. sERP generation

The sERP *y*(*n*) is generated following (2) in Section 2.1. Notice that due to the event type-dependent nature of the SNR estimation for ERPs, the signal *s*(*n*) is first separated according to the event type *k* before zero-mean Gaussian noise $\epsilon_{k}\sim N\left(0,\sigma_{\epsilon_{k}}^{2}\right)$ is added to the sub-signals *s*_*k*_(*n*) to yield *y*_*k*_(*n*)

(26) $$\begin{array}{l} y_{k}\left(n\right)=s_{k}\left(n\right)+\epsilon_{k}\left(n\right). \end{array}$$

The values of $\sigma_{\epsilon_{k}}^{2}$ are determined in dependence on the SNR condition according to (25). After the addition of noise to the sub-signals, the sERP *y*(*n*) is derived by combining *y*_*k*_(*n*) for both event types in their original trial order. We created noise conditions of SNR [dB] ∈ {10, 8, 6, 4, 2, 0} which are reasonable for ERP data (Kolossa, 2016). For each of the *L* = 16 subjects ℓ∈L={1,…,L}, the SNR is drawn from a normal distribution with 2 dB variance ${\text{SNR}}_{\ell }\sim N\left(\text{SNR},\sigma_{\text{SNR}}^{2}=2\text{dB}\right)$ to model variability of SNRs over subjects. For each SNR condition, ten different numbers of data points *N* ∈ {50, 100, 150, 200, 250, 300, 350, 400, 450, 500} are used, yielding a total of 60 scenarios with different combinations of SNR and *N*.

In each scenario, a sequence of *N* events is randomly drawn, with a probability for the frequent event of *P*_*k* = 1_ = 0.7 and for the rare event of *P*_*k* = 2_ = 0.3. The DIF model is used to calculate predictive surprise values which are then degraded by noise as described above in Section 2.1 to yield the sERP. All models *m* of the model space M={NUL,MAR,SQU,DIF} are then subjected to BMS (see Section 2.2). After fitting the models, the Spearman correlation (see Section 2.3) between the DIF model and the MAR model as well as between the DIF model and the SQU model plus the explained variance (see Section 2.4) of the MAR, SQU, and DIF model are calculated. When a scenario is completed for all *L* subjects, exceedance probabilities φ and the median Spearman correlation ρ and explained variance *R*^2^ are calculated to obtain group-level results. Each scenario is simulated five hundred times with new sampling of stimuli and errors (Penny, 2012). Finally, the medians of exceedance probabilities, Spearman correlations and percentages of explained variance over all 500 repetitions are obtained. The following pseudocode summarizes the simulation procedure:


start
for SNR = 0,... ,10 [dB]
   for num. data = 50,... ,500
     for 500 simulations
        for 16 subjects
           SNR sampling
           stimulus sampling
           noise sampling
           Spearman correlation
           expl. variance
           model evidence
        end over subjects
        exceedance probabilities
        median Spearman correlation
        median expl. variance
     end over simulations
     median exceedance probabilities
     median Spearman correlation
     median expl. variance
  end over num. data
end over SNR
end


## 3. Results

**Figure 1:**
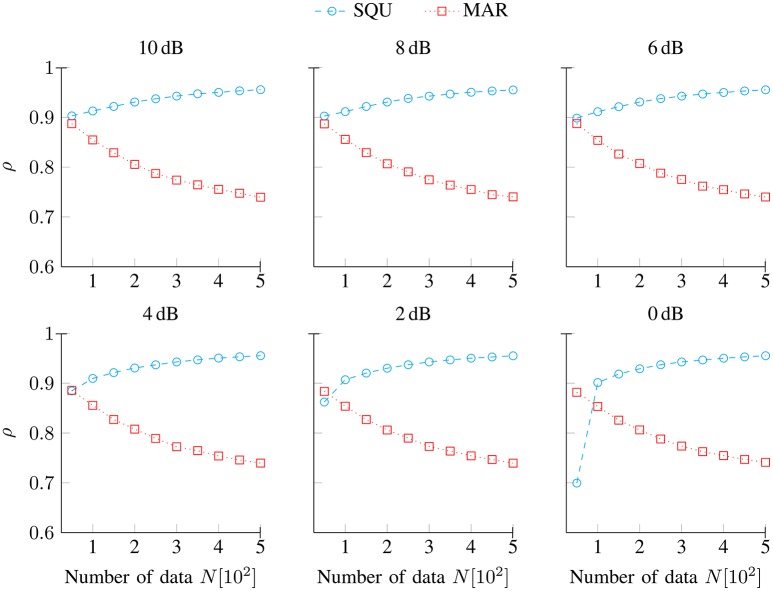
**Spearman correlations ρ (5) between predictors from the data-generating DIF model and predictors from the SQU and the MAR model, respectively**. They are shown as a function of the number of data *N* and separately for noise conditions SNR [dB] ∈ {10, 8, 6, 4, 2, 0}.

Figure 1 shows the median Spearman correlations ρ (5) between predictors from the data-generating DIF model and the SQU model (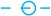) and the MAR model (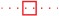), respectively, as a function of the number of data *N* (per individual) and separately for noise conditions SNR [dB] ∈ {10, 8, 6, 4, 2, 0}. Overall, the values of these correlations are quite high, and they are largely independent from variations in data quality (SNR). It is clearly visible that the predictors from the DIF model and from the SQU model were generally much closer correlated than were the predictors from the DIF model and from the MAR model. As an exception from that rule, the DIF model and the SQU model were closer correlated than were the DIF model and the MAR model for the simulations under 2 dB and 0 dB for 50 trials. The DIF–MAR correlation under these circumstances may be attributable to the surprise metric (9) that both models incorporate, while the SQU does not make use of the surprise metric. Thus, for low data quality and low numbers of data points, the shared surprise metric seems to drive the dependency, whereas the model's parameter structure (multifactorial in case of the DIF and SQU models, unifactorial in case of the MAR model) is the stronger determinant of the dependency between the models under all other circumstances. Finally, the dissimilarity between the MAR and SQU model becomes more and more apparent with increasing numbers of data points *N* throughout all SNR conditions.

**Figure 2:**
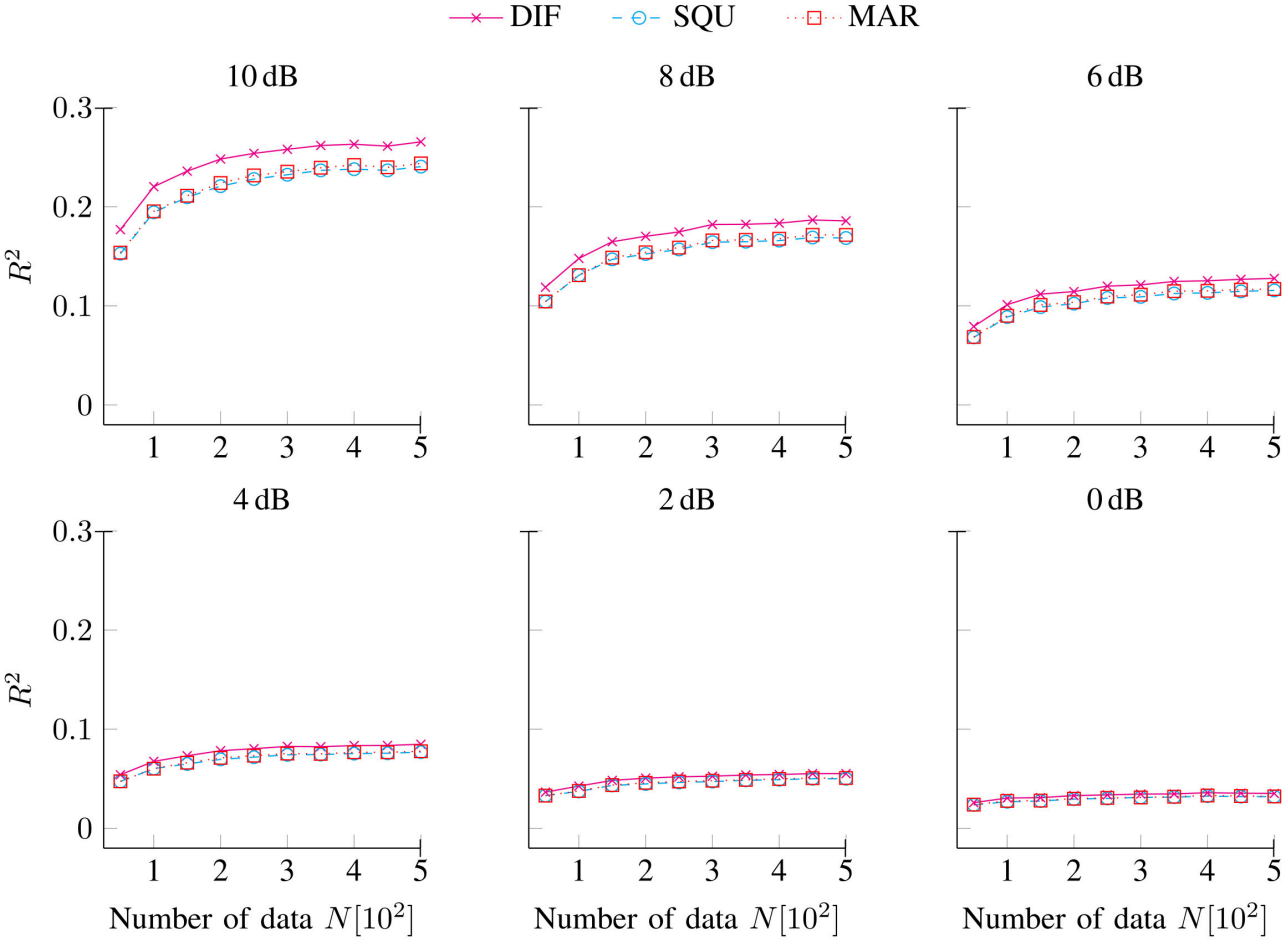
**Explained variance ***R***^**2**^ (6) for the DIF, SQU, and MAR models as a function of the number of data ***N*** separately for noise conditions SNR [dB] ∈ {10, 8, 6, 4, 2, 0}**.

Figure 2 shows the explained variance *R*^2^ (6) for the DIF (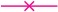), SQU (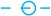), and MAR (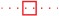) models as a function of the number of data *N* for noise conditions SNR [dB] ∈ {10, 8, 6, 4, 2, 0}. As expected, the amount of explained variance decreased with decreasing SNRs (Penny, 2012). At highest levels of data quality, the maximum amount of explained variance approached around 25%, while at lowest levels of data quality, the maximum amount of explained variance approached less than 5%. Throughout the full range of SNRs and numbers of data points, the data-generating DIF model accounted for the maximum amount of variance. However, the DIF model's superiority in explaining variance decreased with decreasing SNRs. Finally, while the SQU model and the MAR model were clearly dissimilar with respect to their inter-correlations with the data-generating DIF model (see Figure 1), these two models were by-and-large indistinguishable in terms of the amount of variance that they accounted for.

**Figure 3:**
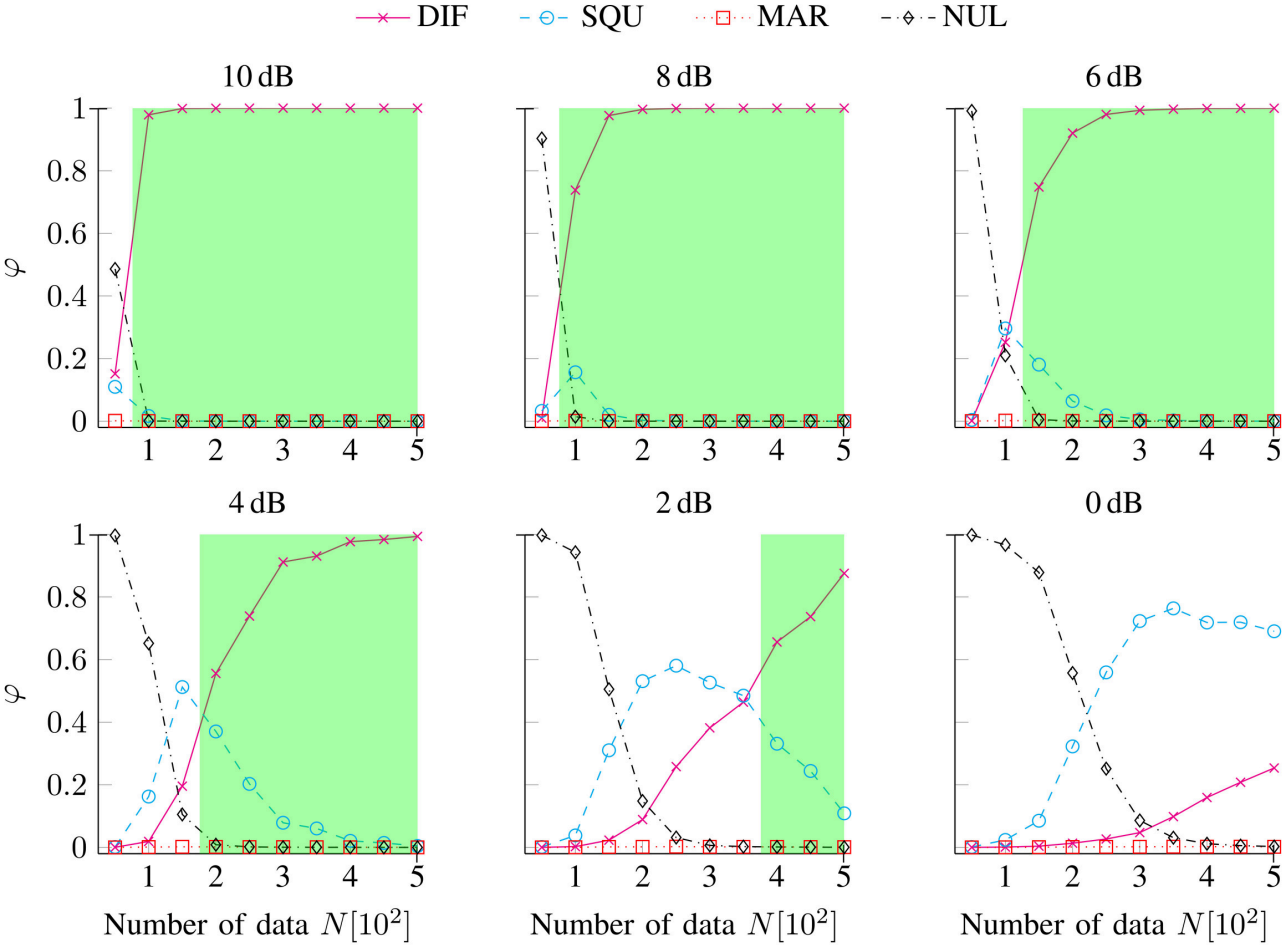
**Exceedance probabilities φ for the DIF, SQU, MAR, and NUL models as a function of the number of data ***N*** separately for noise conditions SNR [dB] ∈ {10, 8, 6, 4, 2, 0}**. The green areas depict the range of valid inference (i.e., the maximum exceedance probability is assigned to the data-generating DIF model) separately for each SNR condition. It can be seen that the range of valid inference shrinks with decreasing SNRs such that no valid inference remains possible at the lowest level of data quality (i.e., at 0 dB) within the given numbers of data points.

Figure 3 shows the exceedance probabilities φ for the DIF (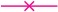), SQU (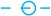), MAR (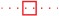), and NUL (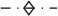) models as a function of the number of data *N* separately for noise conditions SNR [dB] ∈ {10, 8, 6, 4, 2, 0}. At the lowest numbers of data points *N* (between 50 and 200, depending on the SNR), the NUL model achieved maximum exceedance probabilities, while the MAR model never achieved maximum exceedance probabilities. At higher numbers of data points *N*, the data-generating DIF model rapidly achieved superiority for the highest levels of data quality (i.e., SNR condition 10 dB to 6 dB). At the lowest levels of data quality (i.e., SNR condition 4 dB to 0 dB), the SQU model transiently achieved higher exceedance probabilities than did the data-generating DIF model: At a level of data quality of 4 dB, this held true at *N* = 150 trials; at 2 dB, the range of SQU model superiority extended to *N* = 200 to 350 trials; and at 0 dB, the range of SQU model superiority extended to *N* = 250 to 500 trials.

Putatively, the SQU model's superiority across low SNRs and numbers of data points stems from two facts. First, the predictors from the data-generating DIF model and those from the SQU model were highly redundant (see Figure 1). Second, the SQU model incorporates event probabilities, while the data-generating DIF model estimates event probabilities via relative frequencies across trials, and this trial-by-trial variability contributes additional variance to the model's predictors. At relatively low data quality and at low numbers of data points, the PEB scheme probably misattributes this additional variability to noise rather than to the clean signal, rendering the SQU model superior to the data-generating DIF model under these circumstances.

## 4. Discussion

The validity of forward modeling studies in CCN has, in the past, been culpably neglected in the literature albeit that topic is of utmost importance for our ability to identify proper computational models of cognition from studies of brain activity. Here, we showed in a synthetic EEG study that the validity of model selection varies with the data quality, with the numbers of data points, and with complexity and the dependency in the model space. Figure 3 depicts the main findings of our simulation study in terms of exceedance probabilities, a main outcome measure of BMS.

To begin with, the least complex model (i.e., the NUL model) had a competitive advantage at very low numbers of data points throughout the full range of the SNRs that we examined. The data-generating (DIF) model could be easily identified even at relatively low numbers of data points (i.e., around 100 to 200 trials) when SNRs surmounted 7 dB. Below that point of data quality, the SQU model had a competitive advantage over the data-generating DIF model at intermediate (at 2 to 6 dB SNR) or even high (at 0 dB SNR) numbers of data points, such that the paradoxical advantage of the SQU model over the DIF model decreased with rising SNRs.

To summarize, we mis-identified a putative data-generating model at very low numbers of data points at all SNRs (i.e., the NUL model) and throughout intermediate, or even high, numbers of data points as a function of decreasing SNRs (i.e., the SQU model). On the other hand, it is important that we succeeded in identifying the data-generating (DIF) model, provided a sufficient SNRs and/or a sufficient number of data, while a more dissimilar model (i.e., the MAR model) remained less probable than the data-generating model throughout the full range of scenarios. The bottom line from our study is that simulation studies akin to our work should be made mandatory in designing and reporting CCN studies in order to substantiate, rather than merely to suppose, sufficient validity of any given forward modeling study, irrespective of its modality.

One of the basic problems of CCN to date is that the number of data measured in typical forward modeling studies is usually planned without any formal consideration of data quality variations. Consequently, the effects of measurement error remain subject to variation which—as shown—affects the validity of the model selection. Several methods have been suggested for cleaning data (e.g., Turetsky et al., 1989; Effern et al., 2000; Quiroga, 2000; He et al., 2004; Gonzalez-Moreno et al., 2014; Ouyang et al., 2015). However, even though the average data quality can be improved, this does not compensate the insufficiency of the data in potentially many studies. Alternatively, it would also be possible to generally increase the number of data points toward high numbers. In practice, however, fatigue, for example, may affect the neuropsychological phenomena which are under scrutiny (Picton et al., 1995; Boksem et al., 2005; Muller-Gass et al., 2005; Thornton, 2008), and the risks of equipment-related errors also increase with time (Rahne et al., 2008).

Our study provided quantitative results to support the idea that the sufficiency of the number of data points can be better guaranteed by application of a synthetic validity test. For example, the number of data points (*N*_ℓ_ = 1152) in the forward modeling study of Kolossa et al. (2013) was in fact sufficient for selecting between the SQU, MAR, and DIF models, given the empirical data quality (SNR≈2 dB) since inspection of Figure 3 reveals that such a model selection with an SNR = 2 dB falls within the range of valid inference if it is based on *N* > 400 data points per individual. In addition to data quality variations, the model complexity (see the comparison between the NUL vs. the DIF model) and the dependency of the model predictors (compare the comparison between the SQU vs. the DIF model and the comparison between the MAR vs. the DIF model) affect the sufficiency of the number of data points that are required in a valid forward modeling study.

The conductance of a synthetic validity test should incorporate four main variables, i.e., complexity of the models, dependency of the quantitative predictors from the model space, reasonable data quality variations, and feasible numbers of data points (trials). Synthetic validity tests answer the question whether a given number of data points is sufficient or not, given the particular model space under consideration, and given specific assumptions about data quality.

One of our reviewers raised a concern, namely that our explanation why the SQU model (i.e., not the model that was actually used to simulate the data) won the model comparison at very low SNR (and insufficient numbers of data points). We had argued that this can be explained by the way the model works, i.e., in terms of the way the SQU model incorporates event probabilities. An alternative reason why the SQU model might have won our BMS may be related to the hierarchical priors of the PEB approach (Friston et al., 2002; see e.g., Boos et al., 2016 for an example of hierarchical Bayesian modeling). In particular, the prior on the group variance (i.e., the variance of ϵ^(2)^ in 3) might induce more or less “shrinkage around the mean” on PEB estimates, eventually favoring the wrong model in low SNR situations. Thus, the specification of different hierarchical priors at the group level constitutes a variable, which was not explored systematically in our study, but which can be examined in appropriate follow-up studies. In these studies, one could also construct a factorial model space, where DIF, SQU, MAR and NUL would be one model dimension, and different prior variances would induce a orthogonal dimensions. One could then marginalize over prior variances to obtain family-wise exceedance probabilities, which do not depend upon the hierarchical priors (Penny et al., 2010). The same reviewer made the point that exceedance probabilities are but one of many summary statistics in BMS, including posterior estimates of model frequencies and protected exceedance probabilities (Rigoux et al., 2014) that are associated with different levels of statistical risk.

The reviewer also raised the concern that our approach misses a critical aspect of group studies, where data quality refers to the number of trials *N* and data quantity to the number of subjects *L*. Our study does not provide an answer to the question whether one should use, for example, two subjects with 300 trials each (maximizing data quality), or 20 subjects with 30 trials each (maximizing data quantitiy; e.g., Maus et al., 2011). A conceivable extension of our study to evaluate if BMS is more sensitive to data quality or data quantity would be varying (in a factorial way) the within-subject SNR (and/or number of trials per subject) and the group sample size, both chosen within typical ranges. Still another extension would be variations in group heterogeneity (e.g., a group could be composed of individuals best described by different models) which is of particular importance for random-effects BMS. A related issue is the clinical application of BMS, because clinical populations typically differ from normal control populations with regard to SNR (Sackett, 2001; Winterer and Weinberger, 2003), demanding additional strategies for paralleling the SNRs that can be obtained in clinical and normal samples.

We advocated here the idea of using numerical simulations to aid the interpretation of BMS. We showed that one should be cautious about the results of BMS, in case these simulations detect that some of the models may be confused with each other (as is the case for the DIF, SQU, and NUL models here). However, we have not formalized how one would (formally and/or practically) use this information to scaffold one's BMS. In other words: how should one integrate the results of a confusion analysis (derived from realistic numerical simulations) with one's BMS results (performed on experimental data)?

Our idea of conducting a confusion analysis during the design phase of an experiment can be extended to address this issue, as suggested by the reviewer (see Text S1 in Devaine et al., 2014 or Marković and Kiebel, 2016 for examples). To that end, one would derive the full quadratic confusion matrices **C** ∈ ℝ^*M*×*M*^, with *M* denoting the number of models in the model space M. This *M* × *M* confusion matrix yields the exceedence probabilities of having inferred each model, having simulated the data under each model (not just under one of the models, as in our simulation). In such a confusion matrix, the elements on the main diagonal represent the probability of inferring the true (data-generating) model, while the non-diagonal elements represent the probability of inferring a model that did not generate the data. Non-diagnonal elements in this confusion matrix signal potential confusions between the inferred model and the true (data-generating) model; hence, perfect model identifiability should exhibit no extra-diagonal non-zero element, i.e., an identity matrix.

There are many criteria conceivable, which may serve as minimum standards for acceptable levels of model identifiability. An exemplary cut-off criterion may be seen in the requirement that all diagonal elements >0.50 (or alternatively, any other value above 0.50). In this case, however, the chosen cut-off value should strongly depend on the size of the model space. The determinant of the confusion matrix |**C**| may be considered as a more sophisticated approach to quantify model identifiability, because, e.g., |**C**| = 1 for perfect identifiability, while |**C**| = 0 if all models are equally probable. The dependency of the determinant of the confusion matrix on data quality and quantity should be analyzed during a-priori examination of model identifiability, because for any given level of data quantity *L* = *L*′ we get $\left|C\right|{|}_{L\;=\;L\prime ,N\to \infty }=1$, while the value of $0\leq \left|C\right|{|}_{L\to \infty ,N\;=\;N\prime }\leq 1$ depends on the given level of data quality *N* = *N*′. Minimum determinants may be defined as cut-off criteria, with lower limits being associated with less confidence in the conclusions that can be drawn from a forward modeling study. While one may leave the choice of a particular cut-off criterion for acceptable levels of model identifiability to the discretion of the authors, CCN would certainly profit from such an explicit treatment of a-priori model identifiability.

But what if the most plausible model for one's experimental data is easily confused with another model? As far as we know, there are no existing solutions to this issue once the experiment has already been carried out. However, the a-priori calculation of confusion matrices renders it possible to quantify the overall risk of model confusion, which decreases with increasing data quantity and quality. These calculations enable one to conduct a feasible experiment, while controlling for the overall risk of model confusion, as discussed above. However, it may simply not be feasible to collect data with sufficient levels of quality and quantity to surmount the pre-defined cut-off criteria. In this case, the model space may be partitioned into model families, and reasonable amounts of data may suffice for an acceptable confusion risk within the respective model families.

Another solution to this problem would be to strengthen the informativeness of the experimental design, e.g., by applying the technique of adaptive design optimization as proposed in cognitive science (Myung and Pitt, 2009; Cavagnaro et al., 2010, 2011; Myung et al., 2013; Kim et al., 2014). In the context of model identifiability in BMS, adaptive design optimization implies maximizing the determinants of the *M* × *M* confusion matrices under fixed levels of data quality and quantity. This goal can be achieved through the employment of the experimental design that yields the best possible discrimination between model outputs. Engineering solutions for system identification rely on pre-experimental optimization as well, e.g., pseudo random input sequences in non-linear system identification (Billings and Fakhouri, 1980; Vincent et al., 2010) or so-called perfect sequences in acoustic system identification (Ipatov, 1979; Lüke and Schotten, 1995). In the context of CCN, these techniques may be employed to guarantee maximum orthogonality between model outputs, which would enable BMS to better discriminate between the models. Given that these two proposals complement each other, one should first optimize the experimental design, and subsequently analyze minimum levels of data quality and quantity that are necessary for acceptable levels of model identifiability. Those are some of the routes for more systematic efforts toward a-priori calculation of confusion matrices, which may eventually lead to novel solutions to the problem of model identifiability that begins to fan out.

Despite the discussed shortcomings of our work, we recommend for researchers who plan to conduct a forward modeling CCN study, to run an unsolicited a-priori synthetic validity test in order to guarantee sufficiency of the to be gathered data. We further propose that this kind of synthetic validity tests should be made mandatory to all forward modeling studies in the future with the goal to improve the validity of these CCN studies.

## Author contributions

AK and BK conceptualized the study. AK programmed and performed the simulations. AK and BK wrote the manuscript.

### Conflict of interest statement

The authors declare that the research was conducted in the absence of any commercial or financial relationships that could be construed as a potential conflict of interest.
